# Radiologic Predictors for Clinical Improvement in PAO—A Perspective Study

**DOI:** 10.3390/jcm12051837

**Published:** 2023-02-24

**Authors:** Kamil Kołodziejczyk, Adam Czwojdziński, Maria Czubak-Wrzosek, Jarosław Czubak

**Affiliations:** 1 Department of Orthopaedics, Children’s Orthopaedics and Traumatology, Centre of Postgraduate Medical Education, Professor A. Gruca Teaching Hospital, Konarskiego 13, 05-400 Otwock, Poland; 2 Department of Spine Disorders and Orthopedics, Centre of Postgraduate Medical Education, Professor A. Gruca Teaching Hospital, Konarskiego 13, 05-400 Otwock, Poland

**Keywords:** hip dysplasia, PAO, DDH, ilioischial angle, hip joint preservation

## Abstract

The aim of this study was to evaluate the results of surgical treatment of developmental dysplasia of the hip (DDH) with periacetabular osteotomy (PAO) and determine the values of radiological parameters that would allow us to obtain an optimal clinical result. Radiological evaluation included determining the center-edge angle (CEA), medialization, distalization, femoral head coverage (FHC), and ilioischial angle as measured on a standardized AP radiograph of the hip joints. Clinical evaluation was based on the HHS, WOMAC, Merle d’Aubigne–Postel scales and Hip Lag Sign. The results of PAO presented decreased medialization (mean 3.4 mm), distalization (mean 3.5 mm), and ilioischial angle (mean 2.7°); improvement in femoral head bone cover; an increased CEA (mean 16.3°) and FHC (mean 15.2%); clinically increased HHS (mean 22 points) and M. Postel–d’Aubigne (mean 3.5 points) scores; and a decrease in WOMAC (mean 24%). HLS improved in 67% of patients after surgery. Qualification of patients with DDH for PAO should be based on the following values of three parameters: CEA < 26°, FHC < 75%, and ilioischial angle >85.9°. To achieve better clinical results, it is necessary to increase the average CEA value by 11° and the average FHC by 11% and reduce the average ilioischial angle by 3°.

## 1. Introduction

Developmental dysplasia of the hip (DDH) is the result of an abnormal development of the hip joint during the fetal period, affecting both the acetabulum and the proximal femur. Symptoms of DDH may occur in the third decade of life; however, the majority of patients become symptomatic in their fourth decade of life [1,2,3,4]. One of the surgical techniques dedicated to treating DDH is periacetabular osteotomy (PAO), as described by R. Ganz in 1984 [5]. This osteotomy consists of a periacetabular osteotomy of the pubis, ischium, and iliac bones, and it is performed in cases of symptomatic hip dysplasia in skeletally mature patients. Its purpose is the reorientation of the acetabulum to achieve better stability of the dysplastic hip joint. The osteotomy technique leaves the posterior column of the acetabulum intact, which guarantees the stability of the pelvic ring and enables early ambulation [2,3,4,5]. Proper orientation of the acetabular fragment enables the maximum chance of good long-term treatment results. The main goal of PAO is to improve joint congruity and provide better coverage of the femoral head. Reorientation of the acetabulum follows the complete cutting out of the acetabular fragment from the rest of the pelvis. Periacetabular osteotomy is a technically difficult surgical procedure that requires a great deal of knowledge and surgical experience in pelvic surgery. The correcting maneuver leads to improved coverage of the femoral head, medialization, distalization, and reorientation of the entire dysplastic hip joint described as the ilioischial angle [6]. Medialization is a redirection of the acetabular fragment; thus, the entire hip joint moves towards the midline of the body. Distalization involves distal displacement of the subluxated hip joint in the frontal plane. The new position improves the joint’s dynamic stabilization and extends the gluteal muscles lever arm [7,8]. The main objective of this surgical technique is to delay or prevent the development of osteoarthritis, which might lead to total hip replacement surgery [1,3,4,9,10]. Proper qualification and the possibility of appropriate surgical treatment offer a great opportunity to improve the quality of life of a selected group of patients while maintaining their hip joint [11,12]. Proper qualification means avoiding unnecessary exposure to potential intra- and postoperative risks. It also means abandoning PAO in a group of patients with uncertain clinical results.

The aim of this study was to evaluate radiological and clinical outcomes of surgical treatment of DDH with PAO and determine the values of radiological parameters needed to obtain an optimal clinical result. An attempt was also made to assess the correlation of radiological results with clinical results in order to determine more accurate indications for the PAO method in the treatment of DDH.

## 2. Materials and Methods

This prospective study included patients treated in our department with PAO from 2015 to 2018. Inclusion criteria for this study were hip joints not previously surgically treated, symptomatic persistent hip dysplasia, complete and correct radiological documentation from the treatment period, and no degenerative changes in the hip joint (Tönnis grade 0–1) [13]. The study exclusion criteria were secondary acetabular hip, previous surgical treatment, femoral head deformity (joint incongruence, lack of repositioning of the head into the acetabulum on X-ray in hip joint max abduction) and medium or high degree of osteoarthritis of the hip.

Fifty-six patients (fifty-eight hip joints) were included in the prospective evaluation. All patients were included and had surgery performed. The average age was 35.5 years (18 to 48 years). A total of 31 left hip joints and 27 right hip joints were qualified. The group consisted of 51 women and 5 men.

**Figure 1:**
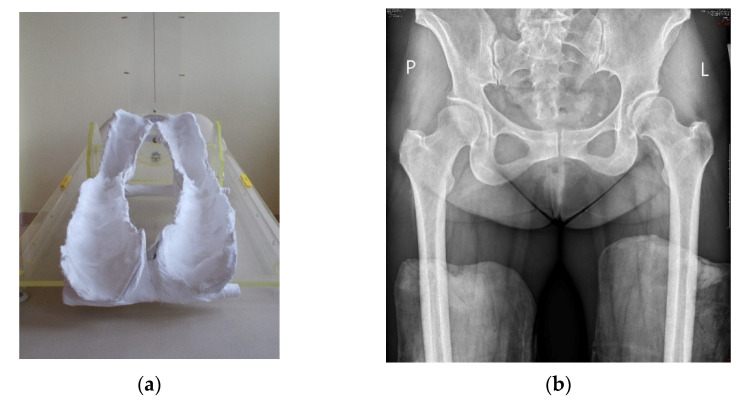
Plaster molds with 20-degree internal rotation of leg (**a**) in which AP digital radiographs were taken (**b**).

The radiological evaluation included standard digital anteroposterior (AP) radiographs of the hip joints performed preoperatively and on the last follow-up. The patient’s position during the examination was standardized: the patient was supine, and the distance between the X-ray machine and the hip joint was 100 cm [14]. Lower limbs were positioned at 20 degrees of internal rotation [15]. To properly position the hip joints in internal rotation, special plaster molds were made with 20° of internal rotation, in which all of the radiographs were taken (Figure 1).

The internal rotation position allowed us to eliminate the natural antetorsion of the femoral neck on the image of the proximal femur [15]. The following radiographic parameters were measured: CEA [16], FHC [17], medialization, distalization and the ilioischial angle [6]. The postoperative acetabular version was assessed on the basis of a crossover sign on the AP digital radiogram of the hip joints.

**Figure 2:**
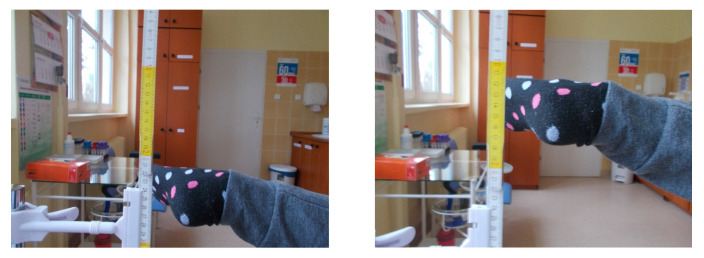
Clinical examination of the hip abductor muscle function Hip Lag Sign test.

Clinical assessment included using multiple scales: the modified Harris Hip Score [18], the WOMAC, and the Merle d’Aubigne–Postel [19]. In addition, age, BMI, and hip flexion range were noted in the study protocol. Gluteal muscle efficiency was tested clinically using the Hip Lag Sign (HLS) [20] test (Figure 2). DDH staging was conducted on McKay’s [21] clinical classification and Crowe’s [22] radiological classification. Radiological and clinical controls were performed before and approximately 4.5 years after surgical treatment. All measurements were taken by a single researcher (orthopedic surgeon).

The study received approval from the Institutional Ethics Committee, number: 83/PB/2015. Informed consent was obtained from all of the patients. Using CareStream Solution software (CareStream Health, Rochester, NY, USA), the accuracy of measurement was determined to be 0.5 degrees for angles and 0.5 mm for distances. The normal distribution was assessed using the Shapiro–Wilk test. For the normally distributed parameters paired *t*-test, and for parameters with an abnormal distribution, the Wilcoxon signed-rank test was used. Dependence between radiological parameters was assessed using linear correlation. The impact of changes in radiological parameter values on improvements in clinical scale scores was evaluated using ROC curves. Significance was set at *p* < 0.05. Analysis of the data included descriptive statistics and was performed in Stata v. 11.0 (StataCorp, College Station, TX, USA) and Excel (Microsoft, Redmond, Washington, DC, USA).

## 3. Results

The average follow-up time amounted to 54 months (48 to 72 months). According to McKay’s criteria, results were classified as follows: good: 21; fair: 19; poor: 18 (Table 1).

The McKay classification confirmed the appropriate selection of patients with symptomatic persistent hip dysplasia. There were no patients in the “excellent” group, who, according to this classification, have no symptoms of hip instability or pain, in the study. Based on the Crowe classification, patients were classified as type I: 30 hip joints and type II: 28 hip joints. The distribution of patients according to the Crowe classification confirmed the appropriate selection of patients with persistent hip dysplasia without femoral head deformity and without interruption of the Shenton–Menard [23] line, i.e., without hip subluxation. None of the patients included in the study who completed follow-up required conversion to a total hip replacement. We observed major complications in 3.4% of cases and minor complications in 8%; intra-articular osteotomy was observed in two patients and neurapraxia of the anterolateral cutaneous nerve of the thigh in five patients.

**Figure 3:**
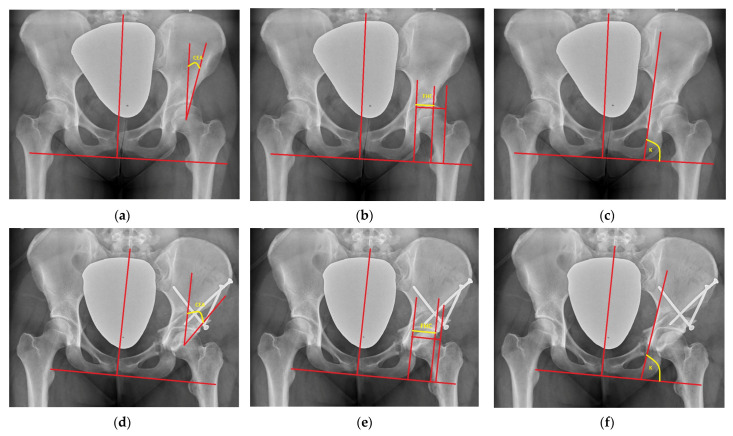
(**a**–**c**) Radiographic measurements of CEA (10°), FHC (52%), and K ilioischial angle (87°) preoperatively; (**d**–**f**) radiographic measurements of CEA (28°), FHC (80%), and K ilioischial angle (82°) postoperatively.

Normal distribution according to the Shapiro–Wilk test was obtained for all radiological parameters, and parametric statistical tests were applied. There was a statistically significant (*p* < 0.05) difference in preoperative and postoperative measurements observed for all of the radiological parameters. Radiological evaluation (Figure 3) revealed that the effect of the surgical correction on the hip joint was decreased medialization by a mean of 3.4 mm (range: 3 to 3.7), distalization by a mean of 3.5 mm (range: 3.2 to 3.8), a decreased ilioischial angle by a mean of 2.7° (range: 2.2 to 3.7), and an increase in the coverage of the femoral head, i.e., an increased CEA, by a mean of 16.3° (range: 12.1 to 20.5) and FHC by a mean of 15.2% (range: 10.8 to 19.8) (Table 2).

**Figure 4:**
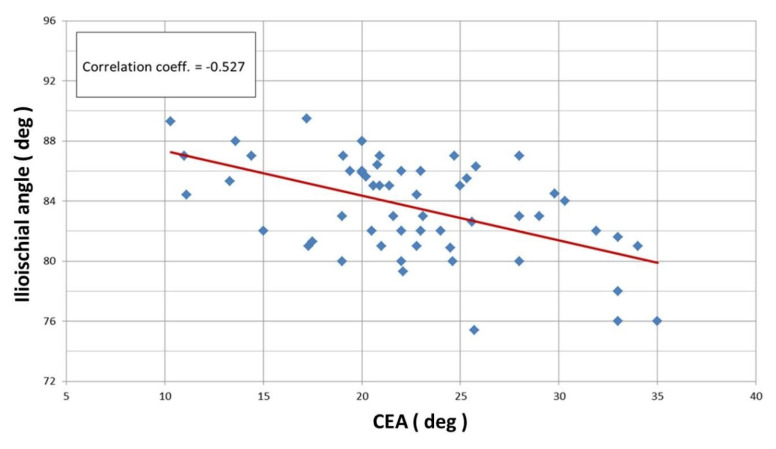
Linear correlation according to Pearson for ilioischial angle and CEA (r = −0.527).

**Figure 5:**
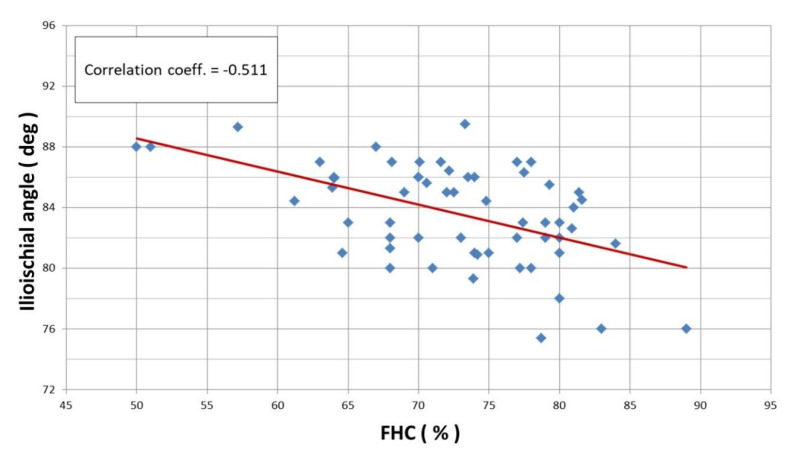
Linear correlation according to Pearson for ilioischial angle and FHC index (r = −0.511).

Linear correlation analysis revealed a statistically significant Pearson correlation between the CEA and ilioischial angle (r = −0.527) (Figure 4) and the FHC and ilioischial angle (r = −0.511) (Figure 5). Based on the crossover sign on the AP digital radiogram of the hip joints, we did not observe acetabular retroversion after PAO in any of the patients.

A normal distribution according to Shapiro–Wilk was obtained for all scale scores, and parametric statistical tests were applied. A statistically significant (*p* < 0.05) difference pre- and postoperatively was observed for all outcomes. The HHS increased by a mean of 22 points (range: 15.8 to 28.2) and the M. Postel–d’Aubigne score by a mean of 3.5 points (range: 2.0 to 4.4), and the WOMAC decreased by a mean of 24% (range: 22.6 to 25.8). Based on the HLS test, an improvement in gluteal muscles efficiency was observed in 67% of patients postoperatively (preoperatively inefficiency seen in 75% of patients, postoperatively inefficiency in 8% of patients). This proves that this new test of gluteal muscle efficiency, i.e., the HLS test, has excellent clinical value (Table 2).

ROC curves were analyzed using statistically significant correlations of linear radiological measurements (CEA, FHC, and ilioischial angle) before and after PAO. Assuming that a good clinical score represents an improvement in clinical scales (HHS, WOMAC, Postel), an optimal clinical score is an improvement by more than the group average. According to prospective data, achieving an optimal clinical score for the HHS scale means an improvement of more than 22 points, more than 24 points for the WOMAC scale, and more than 3.5 points for the M. Postel–d’Aubigne scale. The obtained results of ROC curves and statistically significant results of linear correlation of radiological parameters and clinical scales allowed us to average the improvement in the desired radiological parameters. We could determine with high probability that to obtain an optimal result for all clinical scales, we needed to intraoperatively improve the CEA by 11.7 degrees, FHC by 11%, and the ilioischial angle by 3.3 degrees (Table 3).

The limiting value of the ilioischial angle was calculated analytically based on fitting a mathematical function using the method of least squares to the experimental data. In this case, the fitting function chosen was f(x) = ∛x, which has a tangent perpendicular to the *X*-axis at the point of inflection. In our case, after fitting the function f(x) = ∛x to the experimental data, the equation of the tangent takes the form:

$$g\left(x\right)=-\left(1\right)*\sqrt[{3}]{\left(x-85.9\right)*64}+9$$

**Figure 6:**
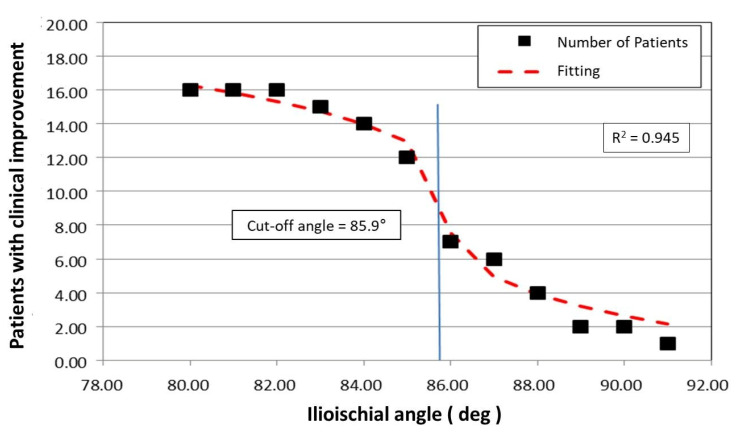
Graphic of the mathematical function that determines the limit value of the ilioischial angle.

The adopted mathematical model indicates the limiting value of the ilioischial angle, x_0_ = 85.95°, with the coefficient of fitting the function to the experimental data, r = 0.945. The limiting value of the ilioischial angle demonstrated by mathematical analysis coincides with clinical observations, which indicates that the greater the ilioischial angle, the more severe the form of dysplasia. The results of linear correlations also show that the higher the value of the ilioischial angle, the lower the values of the CEA and FHC. With the help of a mathematical function, we obtained a cut-off value of 85.95°, which is an additional criterion we can use to qualify patents for surgical intervention. Patients with ilioischial angle values above the cut-off value (85.95°) presented worse results on clinical scales before the surgical treatment (Figure 6).

## 4. Discussion

There are many documented and recognized parameters for assessing hip joint configuration. Many authors suggest the routine use of CT scans as a certainty in the evaluation of hip dysplastic configurations [24,25]. Our observations and studies were based on standardized radiographic images of the pelvis and hip joints in standardized anteroposterior radiographs, as we considered this imaging method to be the most common, cheap, available, and minimally radiologically exposing.

Siebenrock et al. [3] present the results of a group of 75 patients with DDH treated with PAO with a long-term follow-up of 13.5 years. They reported a medialization of 6 mm and noted an improvement in the femoral head coverage CEA of 28°. The mean score on the Merle d’Aubigné–Postel scale before the surgery was about 14.6 points, and after the surgery it was 16.3 points, with a clinical improvement of 1.7 points. Leuning M. and Ganz R. [2] describe the long-term 10-year results of a group of 71 patients with DDH treated with PAO. The authors focused on the presentation of the surgical technique, clinical assessment based on the Merle d’Aubigné–Postel scale, and possible complications of the surgical treatment. They reported a clinical improvement of 1.8 points (preoperative 14.6 points, postoperative 16.4 points). Steppacher S. et al. [4] present an extensive analysis of a 20-year follow-up of patients after PAO. The study group consisted of 63 patients (75 hip joints). The authors noted an improvement in the CEA of 28° (preoperative 8°, postoperative 34°). The authors report preservation of 41 hip joints at 20 years postoperatively (60%). Twenty-six hip joints required total hip arthroplasty (THA). The authors performed a statistical analysis in the form of a Kaplan–Meier survival curve in which the endpoint was joint replacement surgery with a THA. The results are quite promising: a 5-year hip survival rate of about 93%, a 10-year survival rate of 87%, a 15-year survival rate of 77%, and a 20-year survival rate of about 60%. The authors identified risk factors for surgical failure and worse long-term outcomes based on Cox logistic regression. These include an age above 30 years, a preoperative Merle d’Aubigné–Postel score below 14, preoperative degenerative changes above 2 degrees according to Tönnis, and a postoperative hip extrusion rate above 20%. Junfeng Zhu et al. [26] similarly performed radiographic measurements. Their study group consisted of 41 dysplastic hip joints. The follow-up period was 60 months, and the mean age was 39 years. The authors noted a medialization of 6.5 mm. The authors extended the study by measuring the CEA, which averaged 6.4° before surgery and 29.1° after surgery. An improved femoral head coverage of 22.7° was noted. They recorded preoperative HHS scores of 63.7 points and postoperative scores of 88.4 points with a clinical improvement of 24.7 points. Hartig-Andreasen Ch et al. [12] report the overall survival of the dysplastic hip after PAO according to Kaplan–Meier at 74.8% for 12.4 years. The authors suggest that older age, a preoperative Tönnis grade of 2, hip non-congruency and postoperative hip decentration, and a postoperative CEA < 30° or >40° predicted lower hip survival and conversion to THA. These patients should be carefully selected for PAO joint-preserving surgery because they are at an increased risk of poor outcomes. Therefore, preoperative radiographs should be evaluated for the possibility of achieving a good long-term PAO result.

Albers Ch. E et al. [27] include in their clinical assessment the examination of gluteal muscle performance after PAO based on the Trendelenburg sign. The authors divided the patients into two groups: group I (presence of spherical femoral head; n = 43) and group II (absence of femoral head sphericity; n = 122). The authors report a preoperative prevalence of positive Trendelenburg sign of 28% in group I and 20% in group II. At the 10-year postoperative follow-up, the positive Trendelenburg sign was observed in 11% (group I) and 17% of patients (group II). The authors also present clinical results for the Merle d’Aubigne–Postel scale. The results in both groups appear similar. In their conclusion, the authors list risk factors for early hip conversion to THA, which are age >30 years, preoperative Merle d’Aubigne–Postel scale score <15, and advanced osteoarthritis according to Tönnis ≥1-degree grade.

The strength of the gluteus medius and gluteus minimus muscles has a stabilizing effect on the hip joint by pushing the femoral head against the acetabulum [7,8]. HLS is a very useful tool that provides an isolated assessment of the function of the gluteus medius muscle, the primary abductor of the hip joint [20]. Our results show a statistically significant improvement in gluteal muscle function in patients undergoing PAO, resulting in reduced or even no limping in daily life, improved gait efficiency, and reduced strain on the lumbosacral spine and sacroiliac joints.

PAO complications are classified as minor (hematoma, skin conflict over the screws in thin patients), scarring, and neurapraxia of the anterolateral cutaneous nerve of the thigh) and major (femoral nerve damage, vascular damage, uncontrolled intra-articular fracture or damage to the posterior column of the acetabulum, and periarticular ossification). The incidence of major complications ranges from 0.6 and 5.3%, while minor complications range up to 30%. Rare complications include thromboembolism, nonunion, and necrosis of the pelvic acetabular component [10].

## 5. Conclusions

Surgical treatment of DDH with the PAO method shows good results in improving the clinical scales and the function of the gluteal muscles. The radiological improvement in the configuration of the hip joint undergoing PAO is closely related to the improvement observed in clinical results. Increasing knowledge of the relationship between radiological parameters and clinical outcomes involving dysplastic hip allows for a better understanding of the disease and proper qualification for surgical treatment. Proper qualification is certainly the key to the success and maintenance of good long-term results. In the case of borderline hip dysplasia, we recommend the use of three radiographic parameters, CEA, FHC, and the ilioischial angle, in the preoperative evaluation and qualification of patients for PAO.

## Figures and Tables

**Table 1:** Demographic data and classification according to Crowe and McKay.

Number of Patients/Hips	56/58
Sex	Female: 51 Male: 5
Age (mean)	35.5 years (18–48 years)
Operated side	Left: 31 Right: 27
BMI (mean)	23.8
Crowe classification	Type I: 30 Type II: 28
McKay classification	Good: 21 Fair: 19 Poor: 18

**Table 2:** Pre- and postoperative results of hip joint configuration based on radiographic and clinical examination.

Variable (Mean)	Preoperative SD (95% CI)	Postoperative SD (95% CI)	Diff.	*p*-Value
CEA (°)	5.4 ± 8.7 (3.1–7.6)	21.7 ± 7.5 (19.7–23.6)	16.3	<0.0001
Medialization (mm)	83.4 ± 5.4 (81.9–84.8)	80 ± 6.9 (78.2–81.8)	−3.4	<0.0005
Distalization (mm)	53.9 ± 8.2 (51.8–56.1)	50.4 ± 9.4 (48.0–52.9)	−3.5	<0.0005
FHC (%)	57.4 ± 9.2 (54.9–59.8)	72.6 ± 7.2 (70.6–74.7)	15.2	<0.0001
Ilioischial angle (°)	86.5 ± 2.9 (85.7–87.3)	83.8 ± 2.9 (83.1–84.6)	−2.7	<0.0005
HHS (points)	58.8 ± 13.2 (55.3–62.3)	80.8 ± 10.3 (78.1–83.5)	22	<0.0001
WOMAC (%)	35.3 ± 17.9 (30.6–40.0)	11.1 ± 11.8 (8.0–14.2)	−24.2	<0.0001
M. Postel–d’Aubigne (points)	12.9 ± 2.5 (12.2–13.6)	16.1 ± 1.9 (15.6–16.6)	3.5	<0.0001
Hip flexion (°)	120 ± 1.4 (119–122)	118 ± 2.1 (117–122)	2	0.35
HLS (%)	75	8	67	<0.001

Diff, difference; °, degrees; mm, millimeters; CEA, center-edge angle; FHC, femoral head cover; HLS, Hip Lag Sign.

**Table 3:** The results of the desired hip correction according to the parameters of CEA, FHC, and ilioischial angle based on the ROC curve on obtaining a “better” score of the clinical scales HHS, WOMAC, and M. Postel–d’Aubigne.

Variable (Mean)	HHS	WOMAC	M. Postel–d’Aubigne	Improvement for All Scales (Mean)
CEA (deg.)	15.5	10.4	9.4	11.7
FHC (%)	3.6	20	9.2	11
Ilioischial angle (deg.)	3	3	3.9	3.3

deg, degrees; CEA, center-edge angle; FHC, femoral head cover; HHS, Harris Hip Score.

## Data Availability

All data generated or analyzed during this study are included in this published article.

## Abbreviations

DDH	developmental dysplasia of the hip
HHS	Harris Hip Score
HLS	Hip Lag Sign
CT	computed tomography
AP	anteroposterior
CEA	center-edge angle
FHC	femoral head coverage
PAO	periacetabular osteotomy
